# Exploring the Mechanism of Activation of CFTR by Curcuminoids: An Ensemble Docking Study

**DOI:** 10.3390/ijms25010552

**Published:** 2023-12-31

**Authors:** Emanuele Bellacchio

**Affiliations:** Genetica Molecolare e Genomica Funzionale, Bambino Gesù Children’s Hospital, IRCCS, 00165 Rome, Italy; emanuele1b@yahoo.it

**Keywords:** CFTR, cystic fibrosis, cystic fibrosis transmembrane conductance regulator, docking, molecular dynamics, curcumin, CFTR modulators

## Abstract

Curcumin, a major constituent of turmeric (*Curcuma longa* L.), has beneficial effects against several diseases. In cystic fibrosis (CF), this compound improves patients’ symptoms by recovering the activity of a number of mutants of the cystic fibrosis transmembrane conductance regulator (CFTR). Despite holding promise in the treatment of CF, the curcumin binding site in CFTR and the molecular mechanism of activation of this channel are still unknown. The results of this study, based on docking and molecular dynamics (MD) simulations, allow us to propose that curcumin binds the closed ATP-free CFTR near the nucleotide-binding domain 1 (NBD1)/ICl1/ICl4 interface. The bound ligand, once approached by the nucleotide-binding domain 2 (NBD2) during transient channel opening, lays at a multiple interdomain cross point. Thereafter, curcumin can bridge NBD1 and NBD2, and also ICL1/ICL4 and ICL2/ICL3, finally tightening the same interdomain interactions that normally uphold the open conformation in the wild-type ATP-bound CFTR. The proposed binding site is compatible with biochemical observations made in previous CFTR–curcumin interaction studies. These findings provide a framework for the design of novel drugs that activate CFTR mutants characterized by defects in ATP binding and/or NBD dimerization or even lacking NBD2.

## 1. Introduction

The cystic fibrosis transmembrane conductance regulator (CFTR) is a cAMP-dependent member of the large superfamily of ATP binding cassette (ABC) transporters, characterized by four canonical domains, two transmembrane domains (TMDs) and two cytosolic nucleotide-binding domains (NBDs). The main physiologic permeants transported by CFTR are chloride (Cl^−^) and bicarbonate (HCO_3_^−^) anions.

CFTR pore opening and closing (gating) rely on the intramolecular dimerization and dissociation of the NBDs. The former process is promoted by the binding of two ATP molecules, which remain sealed inside the NBD1–NBD2 interface, while the latter follows ATP hydrolysis stimulated by the CFTR intrinsic ATPase activity [1].

**Figure 1:**
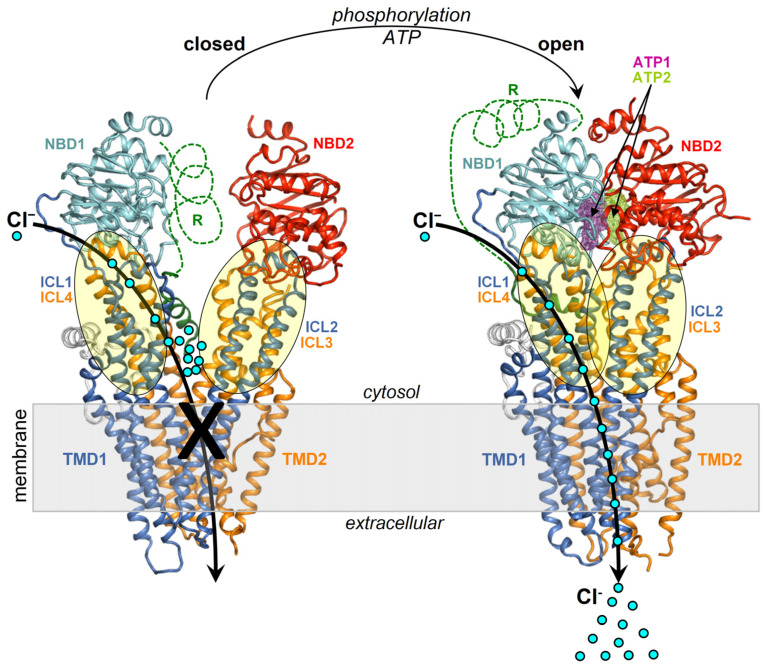
Structures of the closed and open CFTR. Shown are the structures of closed CFTR (Protein Data Base, PDB, 5UAK) and open CFTR (PDB 6MSM), highlighting domains and ICLs with a schematic representation of activation. The protein is coloured by domain (TMD1, blue; TMD2, orange; NBD1, cyan; NBD2, red; other regions in grey).

In the closed channel, the transmembrane (TM) helices are tightly assembled on the extracellular side and gradually spread apart while traversing the membrane and protruding into the cytoplasm as intracellular loops (ICLs) (Figure 1). The ICls are arranged in two distinct intramolecular dimers, ICl1/ICl4 and ICL2/ICL3, which respectively make contact with NBD1 and NBD2. The dimerization of the NBDs promotes the coupling of the ICl1/ICl4 and ICL2/ICL3 dimers into a tetrameric helical bundle, which sparks further rearrangements in the TMDs finally enabling anion transport capability. CFTR also features a regulatory domain (R) that modulates the channel activity in a phosphorylation-dependent manner [2]. NBD1–NBD2 dimerization is prevented by the intramolecular binding of the R domain to NBD1 and the ICLs, and this self-inhibition vanishes along with phosphorylation at various sites by protein kinase A [3,4,5] and protein kinase C (PKC) [6]. These post-translational modifications are thought to weaken the obstructive intramolecular interactions by the R domain. Intriguingly, NBD2 is not strictly necessary for channel opening as CFTR molecules lacking all or most of the domain residues, such as the CFTR-Δ1198 construct, the nonsense mutant CFTR-W1282X [7], and the CFTR-1248X construct [8] present very low yet detectable chloride transport activity. Nevertheless, both NBDs, as well as their dimerizing capability are required for full and ATP-regulated CFTR activation.

Chloride (Cl^−^), the most abundant anion in the organism, exerts various physiological functions. Among these, the substantial transport of this anion across membranes to the cell surfaces, mediated by CFTR, elicits osmosis phenomena moving large water volumes that healthily hydrate epithelia. Therefore, mutations impairing the expression or activity of the CFTR channel are inevitably associated with the onset of viscous secretions abnormally persisting on the epithelia of various organs, especially lungs, pancreas, intestine, and hepatobiliary ducts, which in turn promote bacterial proliferation, infections, obstructions, and fibrosis. These clinical features represent the hallmark of cystic fibrosis (CF), a common autosomal recessive genetic disorder among Caucasians [9]. Although CF is fatal, the life quality and expectancy of patients have improved significantly through the decades owing to more widespread diagnosis and improved symptomatic treatments [10].

In the last few years, a number of small molecule drugs capable of binding and recovering the function of a number of defective CFTR protein mutants, named modulators, were introduced in the clinic and also used in combination [11]. The search for new modulators is still ongoing, and the therapeutic arsenal against CF can also be enriched by various natural substances exhibiting CFTR modulation capability [12,13]. Interestingly, combinations of natural modulators [14] or synthetic and natural modulators [15] can synergistically act against CF, holding the promise of more options to improve the treatment of this disease.

A promising natural CFTR modulator is curcumin, a compound present in turmeric, a spice traditionally used in the Middle East and nowadays diffused around the world. Egan et al. [16] first showed that curcumin corrects functional defects associated with the CFTR-ΔF508 mutation in mice. Subsequently, curcumin efficacy against CF was questioned in the aftermath of a number of contrasting experimental results. Nevertheless, discrepancies among various studies might have been caused by differences in the materials and methods employed, such as the genetics of mouse models, the mutations of patients, the pulmonary functions and responses monitored in the clinics, the treatment duration, and the curcumin source, preparation, storage, and dosing [17]. As a matter of fact, a known major limitation in curcumin use is the difficulty to achieve therapeutic concentrations because this compound is characterized by very poor bioavailability [18]. In a phase I clinical trial, patients with high-risk or pre-malignant lesions who were given curcumin at doses of 4000, 6000, and 8000 mg/day presented, respectively, Cmax values (peak plasma concentration) of 0.51 ± 0.11, 0.64 ± 0.06, and 1.77 ± 1.87 μM [19]. Furthermore, curcumin presents low solubility and rapid degradation in neutral–basic aqueous solutions [20] and tautomerism between the diketo and keto-enol forms with a solvent-dependent equilibrium [21,22]. pH and temperature also regulate this tautomerism, influencing the aggregation propensity and, in turn, the absorption and bioavailability of curcumin [23]. Thus, any change in the experimental setting that may affect the complex molecular properties of curcumin can lead to reproducibility issues in experiments assessing the compound efficacy against CF. Consequently, efforts were made to devise pharmacological formulations that improve curcumin bioavailability through a more efficient delivery of the compound [24]. Notably, a clinical trial showed that children with CF who were administered curcumin nanoparticles presented significant improvement in their quality of life [25].

Concerning the curcumin binding site in CFTR, its location is unlikely to overlap with the site exploited by the synthetic drug ivacaftor, positioned in the TMDs within the lipid bilayer, as the natural and the synthetic modulator potentiate CFTR activity synergistically [15]. Synergistic restoration of the gating defect of G551D-CFTR was observed upon combining curcumin and the natural isoflavone genistein [14]. Interestingly, this synergy was observed in the lower concentration range (5 μM curcumin and 10 μM genistein), while at higher concentrations, the two compounds potentiate CFTR additively, which led the authors to propose that they act through distinct mechanisms. Wang et al. [7] suggested that curcumin interacts with the NBD1 domain following the observation that ATP strongly prevents curcumin-induced stimulation of channels lacking NBD2 and that this inhibition is blunted by the A462F mutation known to disrupt ATP-binding. The same authors also showed that curcumin markedly stimulates poorly active CFTR channels due to defects in ATP-binding and/or NBD1-NBD2 dimerization, such as the CFTR-G551D mutant, and even channels lacking NBD2, such as the nonsense CFTR-W1282X mutant and the CFTR-Δ1198 construct. Yet, these findings allow us to exclude NBD2 as an exclusive binding target in curcumin-induced activation. Wang [26] highlighted two independent curcumin-mediated potentiation mechanisms, one in which the ligand sequestrates the inhibitory Fe^3+^ ions from phosphorylated CFTR and the other in which it binds, as suggested by mutagenesis experiments, to or near ICL1 residues Tyr161 and Lys166, and ICL4 residues Arg1066 and Phe1078. It is worth noticing that all these residues also happen to be near the interface formed by the parent ICLs with NBD1.

In this study, docking and molecular dynamics (MD) were employed to identify a possible curcumin binding site and propose a mechanism for ligand-induced CFTR activation.

A number of docking studies were carried out to understand how various ligands can bind and modulate CFTR. Docking of genistein on a homology model of the NBD1-NBD2 heterodimer led to five putative binding sites with the highest binding affinity predicted for sites 1a and 1b respectively comprising Trp401 and Tyr1219, which are residues important for stable binding of the two ATP molecules in their distinct protein pockets [27]. Docking to a potential binding site using a model of the NBD1/ICL4 interface, thought to be disrupted in CFTR-∆F508 was performed to elucidate the binding mode of the lumacaftor (VX-809) corrector [28,29]. Another docking study employing as ligands lumacaftor and two investigational class II bithiazole correctors, corr-4a and core-corr-II, and a larger CFTR region as the target yielded multiple potential binding sites at various interfaces originating from the contact among the two NBDs and the four ICLs [30]; the same study also determined that the binding energies for the reported sites ranked differently depending on the particular CFTR model employed as the docking target, i.e., full length or ∆NBD2, closed or open, and CFTR-∆F508. A docking study also raised the possibility that lumacaftor binding to NBD1 allosterically modulates the NBD1-ICL4 interface [31]. Molinski et al. [32] proposed through docking that three class I correctors, including lumacaftor, tezacaftor (VX-661), and the investigational compound C18 bind CFTR in a groove comprising Lys166, Tyr380, Thr382, Arg1066, and Gln1071. Docking was also used to guide the design of new analogs of aminoarylthiazole-VX-809 hybrid compounds exhibiting experimentally known ability in correcting CFTR-∆F508 [33,34]. Indeed, shortly later, cryo-electron microscopy (cryo-EM) showed that both lumacaftor and tezacaftor insert their 1-(2,2-difluoro-2H-1,3-benzodioxol-5-yl)cyclopropane-1-carboxamide moiety in a narrow hydrophobic pocket of CFTR composed of cytoplasmic segments of TM helices 1, 2, 3, and 6 while laying the remaining drug portions outside for interactions with protein surface amino acids [35]. Cryo-EM also showed that, differently from lumacaftor and tezacaftor, the type III corrector elexacaftor (VX-445) migrates inside the membrane space and exploits a much shallower binding groove on the CFTR surface contributed mostly by TM helix 11 and to a lesser extent by TM helices 2 and 10, and the lasso motif [36]. Docking normally produces, among the top ranking results, a number of false positives in addition to native-like binding poses. Nevertheless, not all docking-predicted binding sites that are unsupported by cryo-EM can be dismissed as meaningless. Indeed, the mode of binding of CFTR modulators so far disclosed by cryo-EM alone still seem insufficient for a comprehensive understanding of the mechanisms of action of these drugs in CF. Indeed, there are binding sites proposed by docking that are alternative to those captured by cryo-EM but are supported by other experimental techniques. For example, after Liu et al. [37] showed through cryo-EM that ivacaftor (VX-770), the first approved CFTR potentiator, binds CFTR in a cleft formed by TM4, TM5, and TM8 inside the membrane lipidic region, Laselva et al. [38], through a photoaffinity label approach, confirmed the above binding region and also proposed an additional binding site in ICL4 within the region of the ICL tetrahelical bundle. The latter authors pointed out that ivacaftor binding in the two distinct sites might stabilize the open channel either independently or cooperatively through allostery. A computational analysis of the interaction of a library of 220 known type I corrector drugs with five potential binding sites either on the wild-type protein or the CFTR-∆F508 mutant further supported the concept that a given molecule could bind to multiple sites as well as the possibility of promiscuous binding to a same site by different molecules [39]. These studies suggest that ligands might interact in more than one single mode with the protein to produce their biological effects, and not all binding modes can be easily observed experimentally, especially those involving weaker and transient interactions.

## 2. Results

### 2.1. Docking

#### 2.1.1. Working Hypothesis, Ligands and Protein Targets

**Figure 2:**
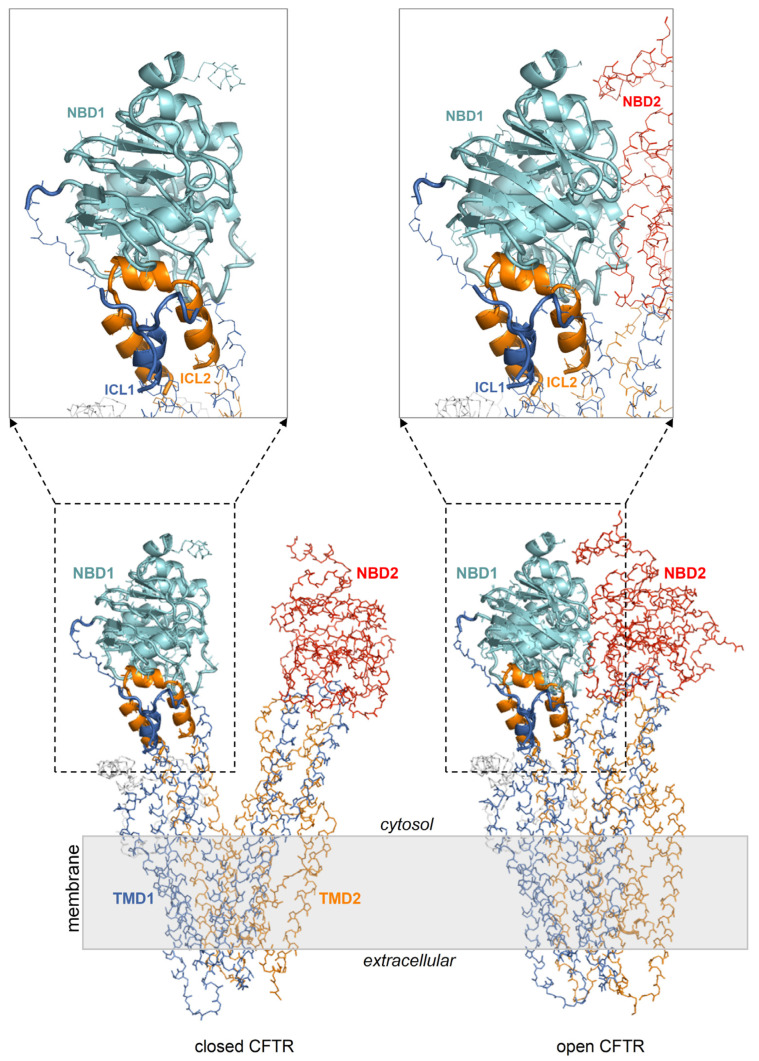
NBD1/ICL1/ICL4 is conformationally invariant in the closed and open CFTR. Closed and open CFTR (respectively, PDB 5UAK and 6MSM, represented as backbone lines with the domains in different colors; the solved R domain fragment and bound ATP molecules are not shown). The conformationally invariant regions (highlighted by cartoons) are defined as those whose C^α^ atoms overlap within 2.5 Angstroms in the closed and open CFTR protein structures superposed relative to NBD1.

It is unknown whether the mechanism for curcumin-induced activation implies, as the initial binding target, the closed or the opened CFTR. The ATP-free active channel is rare and short-lived and can only achieve scarce physiological concentrations. It seems unlikely that this CFTR form can encounter curcumin, which also exhibits very low concentrations. Instead, assuming that curcumin has a comparable affinity for the closed (inactive) and open (active) CFTR conformation, it would be more reasonable that the inactive channels are the initial targets because they constitute the prevalent CFTR fraction and thus, their complexation is favored by the law of mass action. Therefore, it can be hypothesized that curcumin first binds the inactive CFTR protein, and once the channel opens spontaneously, the ligand also stabilizes the active conformation. A simple scenario would be the exploitation of a binding site that is available on both the closed and open CFTR, regardless of the important conformational differences in the two protein states. Indeed, upon comparing the closed and open CFTR, it can be seen that the conformation of the entire NBD1 with the interfacial ICL1/ICL4 portions is essentially invariant (Figure 2). Thus, these regions may host a conformationally conserved site capable of holding a bound curcumin molecule despite the protein shuttles from the inactive to the active state. Coincidentally, a curcumin binding site within NBD1/ICL1/ICL4 would also be in agreement with previously proposed binding regions, i.e., NBD1 [7] and ICL1/ICL4 residues located near the interface of these ICLs with NBD1 [26].

To explore the above hypothesis of a curcumin binding site available on both the closed and open CFTR, the author performed docking searches encompassing NBD1 and the portions of ICL1/ICL4 interfacial with this domain. Even though the NBD1/ICL1/ICL4 region is conformationally stable, it must be taken into account that upon channel opening, the two NBDs, as well as ICL1/ICL4 and ICL2/ICL3, undergo dimerization. Thus, on the cytoplasmic side, the active CFTR protein features a more extended interdomain interface contributed by the two peptide triads NBD1/ICL1/ICL4 and NBD2/ICL2/ICL3 (Figure 1). Consequentially, while most binding poses will be similarly predicted in the closed and open CFTR if the docking target is represented by the isolated NBD1/ICL1/ICL4 region, some of the poses in the open CFTR may indeed be hindered or stabilized by the proximal NBD2/ICL2/ICL3. Furthermore, in the open conformation the protein interface between NBD1/ICL1/ICL4 and NBD2/ICL2/ICL3 could undergo ligand-induced rearrangements, especially in flexible loops. Thus, ligand binding predictions at the dimerization interface between the two peptide triads may lead to unrealistic results if the protein is treated rigidly. To reduce this potential bias, the author reproduced protein flexibility (both side chains and backbone) by employing ensembles of CFTR protein conformers as docking targets.

#### 2.1.2. Aggregated Top-Scoring Curcuminoid Docking Results on Distinct CFTR Ensembles

**Figure 3:**
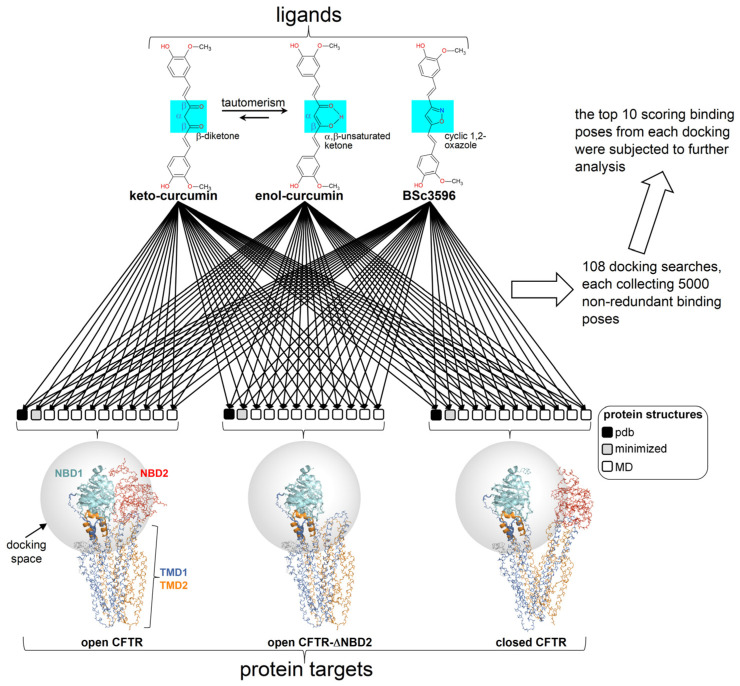
Docking scheme. Curcuminoid structures (diketo curcumin, keto-enol curcumin, and BSc3596) employed in docking (the different chemical moieties in the linkers of the 2-methoxyphenol groups are highlighted by the azure boxes) and CFTR protein targets (ensembles of conformers of the open CFTR, open CFTR-ΔNBD2 construct, and closed CFTR; each ensemble included a parent PDB structure, PDB 6MSM for the open CFTR and the open CFTR-ΔNBD2, and PDB 5UAK for the closed CFTR, the energy minimized and ten MD simulation snapshots of the parent PDB structure; see Section 4). The parent PDB structures are portrayed and colored by the domain (solved R domain fragment and ATP are not shown). The conformationally invariant regions (showing the same conformation in the closed and open CFTR; please see the text) are highlighted by ribbons, and the remainder of the structures by backbone atom lines. The space covered by the docking searches (transparent spheres) encompassed 33.0 Angstroms from the NBD1 center of mass.

Multiple docking searches of three curcuminoid structures (diketo curcumin, keto-enol curcumin, and BSc3596) were launched on three multiple conformer CFTR ensembles representing closed CFTR, open CFTR, and open CFTR-ΔNBD2 construct (each ensemble contains twelve conformers as schematized in Figure 3).

**Figure 4:**
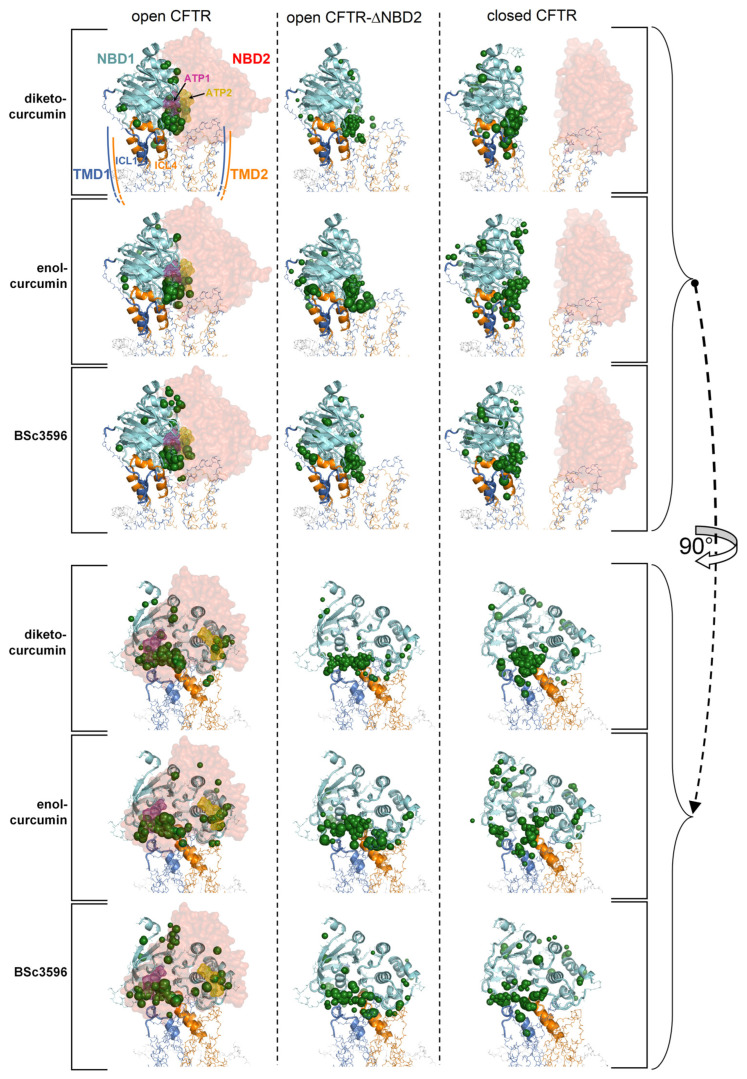
Top-scoring curcuminoid docking results on multi-conformer ensembles of the open CFTR, open CFTR-ΔNBD2, and closed CFTR. Shown are the aggregated top-scoring docking results of the individual ligands on each CFTR multi-conformer ensemble (for each of the twelve protein structure targets comprised in a CFTR ensemble, ten top-scoring docking results, out of the ranked 5000 ligand binding poses were kept; subsequently, all groups of ten top-scoring results from each of the twelve docking searches were aggregated). The top-scoring docking results are represented by green spheres (drawn on the centers of docking poses with radii proportional to the normalized docking scores). For simplicity, only the parent PDB protein structures used to derive the CFTR protein ensembles are shown (PDB 6MSM for the open CFTR and open CFTR-ΔNBD2; PDB 5UAK for the closed CFTR; the solved fragment of the R domain is not shown; the two ATP binding sites are highlighted by magenta and dark-yellow meshes in the open CFTR structure).

To highlight interaction hot spots of curcuminoids on CFTR, the ten top-scoring docking results for the individual ligands on each of the twelve protein structures comprised in a CFTR conformational ensemble were aggregated and plotted on the parent CFTR structure as ligand-centered spheres with radii proportional to the normalized docking score (Figure 4). It can be seen that each curcuminoid presents a main cluster of top docking results near the interface of NBD1, ICl1, and ICL4, partially overlapping with the closer of the two ATP-binding regions.

#### 2.1.3. Identification of a Consensus Binding Mode of Curcumin Available in the Closed and Open CFTR

**Figure 5:**
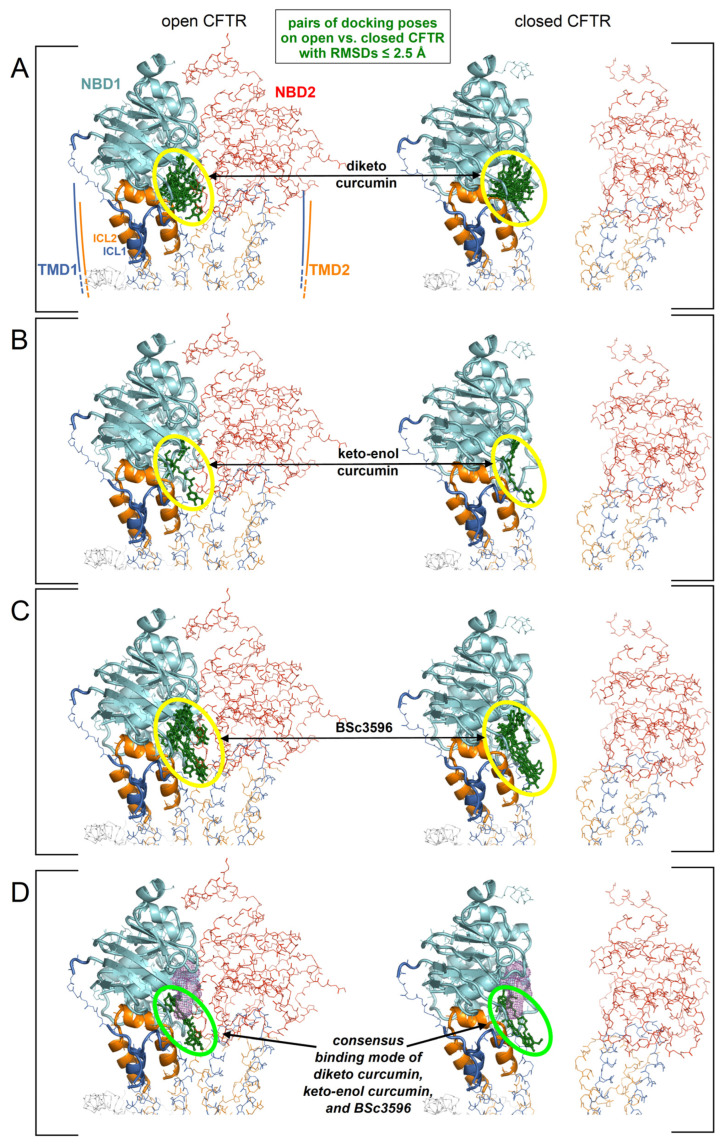
Identification of a consensus binding pose on the closed and open CFTR ensembles. Shown are the aggregated top binding poses of curcuminoids after applying the criterion that the same binding mode must be found on both the closed and open CFTR conformer ensembles (binding poses are shown as green sticks highlighted by the ovals): (**A**) diketo curcumin; (**B**) keto-enol curcumin; (**C**) BSc3596; (**D**) binding poses from panels (**A**–**C**) obeying the additional criterion of binding similarity between curcumin (any of the two tautomers) and BSc3596: only one binding pose fulfills both criteria (and both curcumin tautomers adopts that pose). The displayed protein structures are the open CFTR (PDB 6MSM) and the closed CFTR (PDB 5UAK). The ATP nearest to the consensus curcuminoid binding site is indicated by the magenta meshes in the two bottom protein structures.

The aggregated top-scoring binding poses clustered primarily near the NBD1/ICl1/ICL4 interface, although a significant number of poses were also dispersed elsewhere around NBD1. To reduce the multiplicity of the top-scoring docking results in order to seek a plausible and possibly unequivocal curcuminoid binding site, the author assumed, as hypothesized above, that curcuminoids adopt the same binding mode on both the closed and open CFTR. To this end, the RMSDs between all aggregated top ten scoring binding poses on the closed and the open CFTR ensemble were iteratively calculated (as explained in the legend of Figure 4). In this process, a binding pose was considered to be shared by the closed and open CFTR if it was present at least on one protein conformer in each of the respective ensembles and overlapping within an RMSD of 2.5 Angstroms. By applying this filter, only a small fraction of the top binding poses in the cluster near the NBD1/ICL1/ICL4 interface was retained, and all poses outside this cluster were rejected (Figure 5A–C). The number of binding poses shared by diketo curcumin on the open and closed CFTR ensembles was significantly greater than those of keto-enol curcumin and BSc3596, which can be attributed to the different ligand flexibilities. In fact, compared to diketo curcumin, the keto-enol tautomer has a more extended pi-electron conjugation and also an intramolecular hydrogen bond, both inducing coplanarity of the molecule central atoms and a linear molecular shape [21]. With respect to diketo curcumin, also BSc3596 is constrained into a linear geometry owing to the 1,2-oxazole group. Thus, among the three curcuminoids examined in this study, diketo curcumin is the most flexible and capable of sampling more diverse binding modes inside a protein cavity. Next, considering that curcumin and BSc3596 are chemical analogs and both function as CFTR activators, it can be reasonably assumed that the two ligands activate the channel by exploiting the same binding mode. Despite the fact that it is unknown whether diketo curcumin or keto-enol curcumin is the most active tautomer, it should be safely expected that the native docking of at least one of the two is similar to that of BSc3596. Remarkably, comparing the filtered top-scoring docking results of the three ligands shown in Figure 5A–C, only one binding pose turned out to be shared by the synthetic modulator and at least one curcumin tautomer (indeed, it was shared with both diketo and keto-enol curcumin) (Figure 5D).

**Figure 6:**
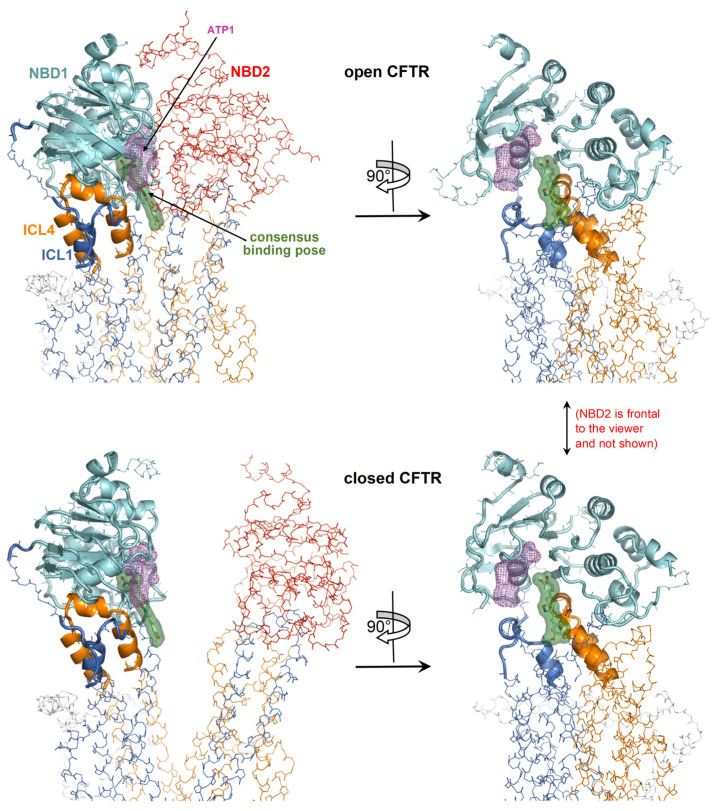
Detailed view of the consensus binding pose. The consensus binding pose of a curcuminoid (sticks and green surface) is shown on the closed CFTR (PDB 5UAK) and open CFTR (PDB 6MSM). The protein is coloured by domain (TMD1, blue; TMD2, orange; NBD1, cyan; NBD2, red; other regions in grey). The protein regions with invariant conformation in the closed and open CFTR (please see the text) are represented by ribbons, the other regions as backbone atom lines. The position of the ATP cofactor in the binding site nearest to the curcuminoid consensus binding pose is indicated by the magenta meshes. The NBD2 domain in the rotated views on the right is in front of the viewer and, for clarity, is not shown.

A curcuminoid in the consensus binding pose occupies a site partially overlapping with one of the two ATP binding sites (Figure 6); thus, there would be binding competition between curcumin and the cofactor, which is consistent with the observation that ATP inhibits curcumin-induced CFTR stimulation.

### 2.2. Molecular Dynamics Simulations

#### 2.2.1. MD Simulations of Curcuminoids Bound to the Open and Closed CFTR

**Figure 7:**
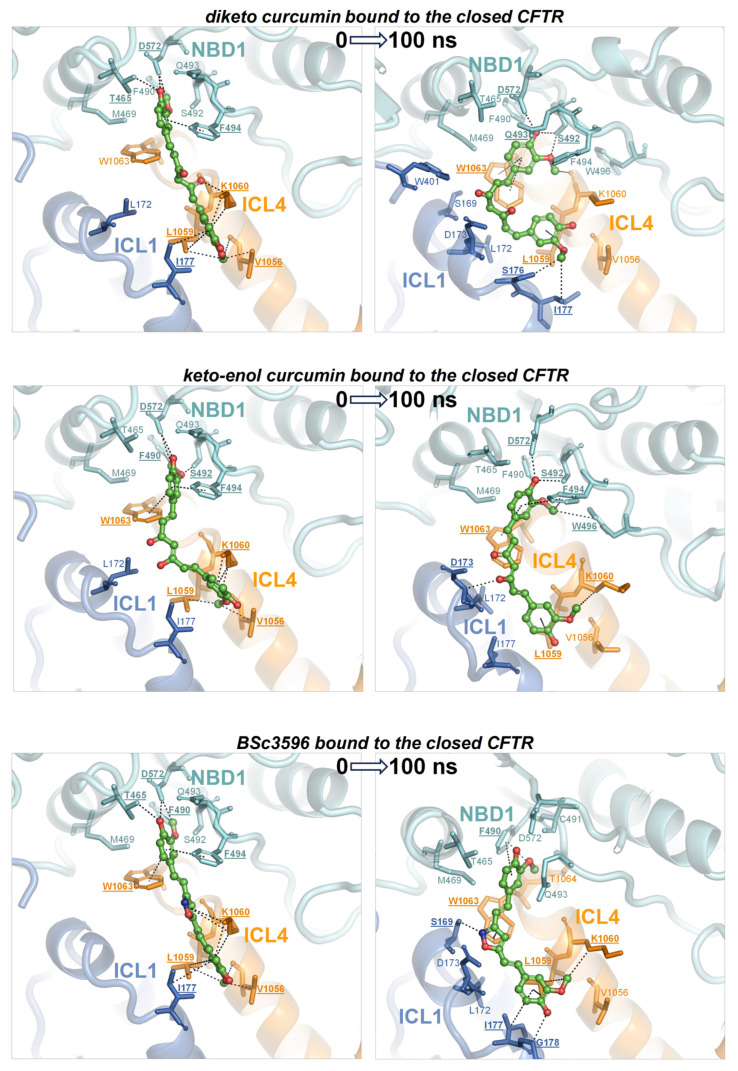
MD simulations of curcuminoids complexed with the closed CFTR. Structures of the three curcuminoids placed in the consensus binding site of the closed CFTR used to kickstart the MD simulations and the same complexes after 100 ns of simulation. The protein is coloured by domain (TMD1, blue; TMD2, orange; NBD1, cyan; NBD2, red). The protein residues near the ligands are shown as sticks (the same color as the parent domains); residues participating in non-bonding interactions (dotted lines) with the ligand are labeled in bold and underscored; other nearby residues requiring only minor movements to engage in interactions with the ligands (as judged by visual inspection) are labelled normally. The ligands are represented as balls and sticks (carbon atoms are in green, oxygens in red, and nitrogens in blue).

**Figure 8:**
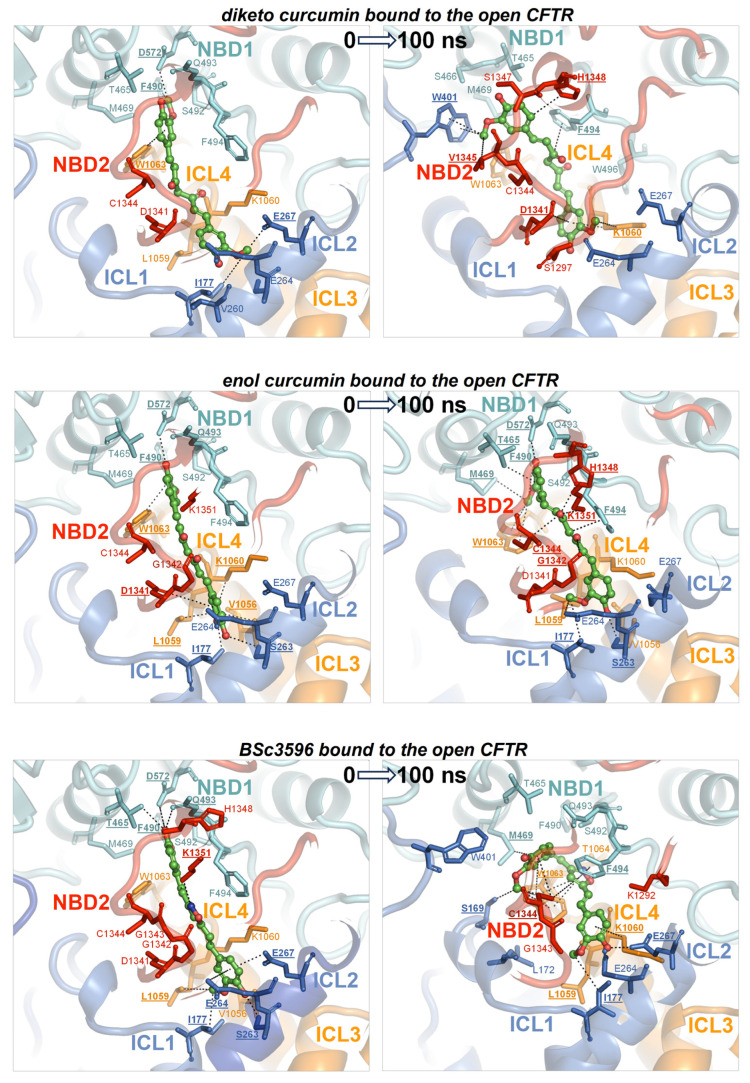
MD simulations of curcuminoids complexed with the open CFTR. Structures of the three curcuminoids placed in the consensus binding site of the open CFTR used to kickstart the MD simulations and the same complexes after 100 ns of simulation. For details on protein/residue colors and labels, please refer to the legend in Figure 7.

To determine whether the consensus binding pose shown in Figure 6 was stably maintained under MD simulations, each curcuminoid was accordingly placed on the closed and open CFTR (respectively, electron microscopy protein structures PDB 5UAK and 6MSM), and the complexes were subjected to 100 ns long MD runs in explicit water solvent. The stability of the molecular systems was monitored using RMSD inspection (no significant drifts were present) (Figure S1). The ligands and surrounding protein residues in their complexes with the closed CFTR structure used to kickstart the MD simulations and the same complexes at 100 ns simulation time are shown in Figure 7. It can be seen that in every curcuminoid complex with the closed CFTR, the ligands remained attached to the initial binding site across the entire simulation length with minimal or modest rearrangements. This supports the idea that curcuminoids can stably linger on the NBD1/ICL1/ICL4 interface in the closed CFTR. Concerning their binding to the open CFTR, it can be seen that the three curcuminoids also remain stably located in the initial site, which in this case turns out to be the cross-point of NBD1/ICL1/ICL4 and NBD2/ICL2/ICL3 (Figure 8).

#### 2.2.2. MD-Derived Energies of Interaction between Curcuminoids and the Distinct CFTR Protein Regions Forming the Consensus Binding Site

**Figure 9:**
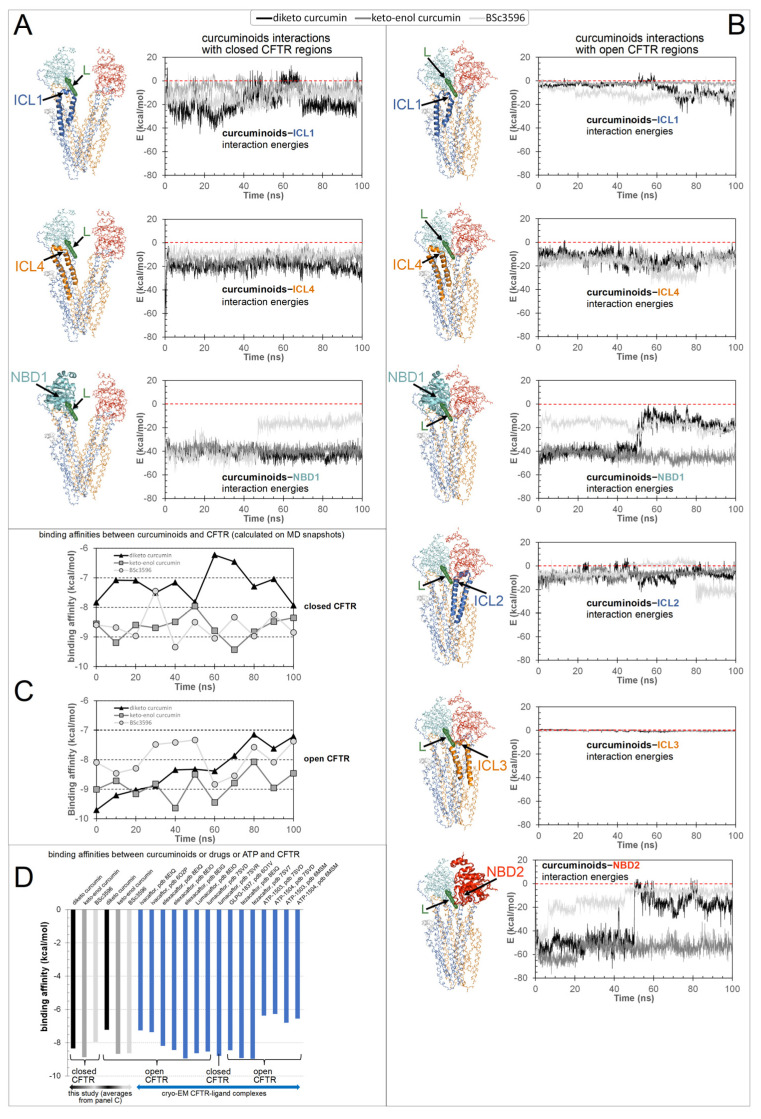
Energies of interaction and binding free energies. Shown are the interaction energies between the curcuminoids (diketo curcumin, keto-enol curcumin, and BSc3596) and CFTR (decomposed in the individual contributions by the ICL1, ICL4, NBD1, ICL2, ICL3, and NBD2 protein regions) calculated on MD simulation snapshots (the zero-energy value is highlighted by red dashed lines). (**A**) Interaction energies between curcuminoids and the open CFTR. (**B**) Interaction energies between curcuminoids and the closed CFTR. The individual protein regions employed in the interaction energy calculations are indicated by an arrow and highlighted with ribbons (the rest of the protein is in backbone lines) in the molecular structure next to each energy plot. The protein is colored by domain (TMD1, blue; TMD2, orange; NBD1, cyan; NBD2, red). The consensus binding site position is indicated by a curcuminoid ligand (L, green meshes). (**C**) Binding affinities between the curcuminoids and the full CFTR protein calculated on MD snapshots taken every 10 ns of simulation (the time 0 ns corresponds to the energy-minimized structure kickstarting the MD simulations). (**D**) Calculated binding affinities between the full CFTR (open and closed) and the curcuminoids (averages from panel (**C**)) or drugs or the two ATP cofactors (as bound in their cryo-EM complexes with CFTR available in the PDB database).

To explore in detail how the various CFTR regions concur to the binding of curcuminoids, the energies of interaction between the ligands and the protein, decomposed in the contributions from the individual ICLs and NBDs, were calculated on MD snapshots across the simulations (Figure 9). In the closed CFTR, the curcuminoids maintained simultaneous interactions with NBD1, ICL1, and ICL4. In the open CFTR, while keeping contact with the same three regions, the ligands also engaged in interactions with NBD2, ICL2, and, to a minimal extent, ICL3. Thus, a salient characteristic of the consensus binding site is that curcuminoids can simultaneously interact with both NBDs and nearly all four ICls. Figure 9C shows the binding free energy (or binding affinity) between the CFTR protein and curcuminoids calculated from MD snapshots taken every 10 ns. Among the two curcumin tautomers, the keto-enol curcumin exhibits a stronger affinity. The binding affinity averages are reported in Figure 9D, together with the binding affinities of drug modulators and ATP calculated on their complexes with CFTR available from PDB cryo-EM structures. It can be seen that the binding affinity of curcumin is comparable with that of CFTR modulators actually used in the clinic.

## 3. Discussion

This study attempts to elucidate the mechanism through which curcumin opens CFTR, bypassing the formation of complexes between the channels and ATP cofactors. This natural compound has a proven capability to activate a number of CFTR mutants with defects in ATP binding and intramolecular dimerization of the NBDs or even lacking NBD2 [7]. To understand how curcumin accomplishes ATP-independent CFTR activation, it is necessary to identify the binding site in the protein and also to know whether the mechanism involves the first binding event to the closed CFTR or already open ATP-free CFTR. It is arguable that curcumin directly binds ATP-free open CFTR to subsequently stabilize it because this type of channel species forms only rarely and transiently and exists at very low levels. Also, curcumin achieves only scarce physiological concentrations owing to very poor bioavailability. Thus, it seems highly improbable that curcumin and the ATP-free open CFTR channels can both be present in the organism at concentrations that allow for the sufficient formation of complexes with a pharmacologic outcome. Nevertheless, the beneficial effects of curcumin in CF can be observed in vivo. A more plausible candidate as the initial binding target of curcumin is the closed CFTR channels, whose concentration is orders of magnitude higher than that of spontaneously activated ATP-free channels. Following binding to the closed CFT, a foreseeable mechanism would be that curcumin promotes CFTR conversion to the active state or, alternatively, that the compound remains bound to the closed CFTR until spontaneous channel opening and subsequently stabilizes the active conformation. Both possibilities would require the presence of a binding site that stably holds curcumin in both closed and open CFTR. The docking strategy presented in this study led to the identification of a binding site near the NBD1/ICL1/ICL4 interface and available in the two CFTR conformations. In the closed channel, the site has the characteristic of a solvent-exposed groove; hence, it must produce sufficient attractive non-bonding interactions to avoid the loss of the ligand. In the open channel, the pose of curcumin on NBD1/ICL1/ICL4 is similar, but the ligand is also sandwiched between this triad and NBD2/ICL2/ICL3. In the latter conformation, the ligand might remain entrapped in the protein cavity since it has no easy escape route to the bulk solvent. Nevertheless, the ligand could undergo rearrangements in both closed and open CFTR, as suggested by the several non-overlapping top-scoring binding poses produced by the docking search near the NBD1/ICL1/ICL4 interface prior to applying the filtering procedures (Figure 4). Furthermore, in the open CFTR, the curcumin molecule might be displaced by conformational changes in the interdomain interface comprising the binding site, which is a likely possibility since this interface is also contributed by flexible loops. Either poor binding affinity for the closed CFTR or curcumin rearrangements in both closed and open CFTR would disprove the above-made hypotheses and the docking procedure. Thus, to assess the plausibility of the consensus binding mode and identify the possible mechanism of channel activation, the author performed MD simulations of the curcuminoids bound to the closed CFTR and open CFTR. At the end of the simulations of the complexes with the closed CFTR, all curcuminoids were still bound (Figure 7). The 2-methoxyphenol groups show engagements in bipartite interactions with two protein patches; one constituted mainly by NBD1 residues plus Trp1063 in ICL4 and the other by ICL1 and ICL4 residues. The linkers between the two 2-methoxyphenol groups also interacted with ICL1 and ICL4 residues. Keto-enol curcumin and BSc3596 maintained their somehow linear shape, whereas the more flexible diketo curcumin underwent significant bending yet still maintained the interactions with the two protein patches. Of note, NBD1/ICL1/ICL4 is enriched in residues producing favorable contact with both the 2-methoxyphenol groups and the different linkers in the three curcuminoids. It also appears that the ligands could experience alternative multivalent favorable interactions, allowing substantial rearrangements within the same site, as indeed was also denoted by the many distinct top-scoring binding poses assigned nearby by the unfiltered docking (Figure 4). In the case of the complexes with the open CFTR, the curcuminoids were also still stably bound to the protein at the end of the MD simulations (Figure 8). Interestingly, the ligands maintained nearly the same pose as in the structures kickstarting the simulations. Unraveling a possible CFTR activation mechanism from these particular ligand–protein geometries would support the plausibility of the same binding model. To this end, the authors examined the energies of interaction between the curcuminoids and the individual protein regions comprising the consensus binding site in MD snapshots across the 100 ns long simulations. It can be seen that NBD1, ICL1, and ICL4 all maintained simultaneous favorable interactions with the curcuminoids bound to the closed CFTR, and, remarkably, in the open CFTR, while keeping the above interactions, also ICL2, ICL3, and NBD2 concurred with favorable interactions (Figure 9). Thus, in the open CFTR, the curcuminoids undertook multiple simultaneous interactions with all the above peptide regions (only ICL3 contributed minimally).

The intramolecular dimerization of the NBDs is not strictly required for channel opening [7]. Nevertheless, this interdomain association is crucial for the implementation of the ATP-dependent regulation in CFTR. In fact, ATP binding strengthens the NBD dimerization, which in turn works as a lever aiding the coupling of ICL1/ICL4 with ICL2/ICL3. The latter process is crucial as it triggers further rearrangements in the TMDs that finally enable chloride transport. It can be reasonably assumed that the spontaneous activation of channels lacking NBD2 involves similar conformational changes in the TMDs with the difference that the activated ΔNBD2 channels are incapable of self-stabilization through NBD dimerization and hence are short-lived. The simultaneous interactions of the curcuminoids with the various regions comprising the binding site in the open CFTR (Figure 9B) allow us to propose that these ligands stabilize this conformation by bridging NBD1/ICL1/ICL4 and NBD2/ICL2/ICL3 once these protein groups approach each other by random domain motions. Such motions might be similar to those normally occurring in the rare spontaneous ATP-free CFTR opening with the difference that the curcumin molecules pre-loaded onto channels will eventually stabilize the association of NBD1/ICL1/ICL4 with NBD2/ICL2/ICL3 (or ICL2/ICL3 in NBD2 deletion mutants) and consequentially the active conformation. Based on the simultaneous favorable interaction energies with NBD1, ICL1, ICL4, NBD2, ICL2, and ICL3 (Figure 9B), curcumin can be viewed as an agent that pulls together these distinct regions, thus stabilizing the NBD dimer and the ICL tetrahelical bundle. In this model, curcumin acts by reinforcing the same interdomain associations that normally sustain the activation of ATP-bound wild-type CFTR channels. In this way, curcumin can compensate for defective ATP-binding, NBD1-NBD2 dimerization, and even NBD2 absence.

Despite the fact that no atomistic level structural information on curcumin interactions with CFTR is available to date, biochemical studies provided hints on the possible binding location for this natural compound. Wang [26] showed that mutagenesis of Tyr161, Lys166, Arg1066, and Phe1078 prevented the Fe^3+^-independent curcumin-induced CFTR activation. Although the same author proposed that Lys166 and Arg1066 may form cation–pi interactions with curcumin aromatic rings, the results of the present study failed to support a direct contact between curcumin and any of the above four residues. Nevertheless, the four mutagenized amino acids are important for the correct assembly of the ICL1 and ICL4 portions comprising the putative curcumin binding site proposed in the present study. Thus, the impairment of CFTR response to curcumin observed in the mutagenized channels might have rather resulted from the indirect abrogation of curcumin binding by the mutation-induced conformational changes in the two ICLs.

Although the calculated binding affinities of curcuminoids and known CFTR-modulating drugs are comparable (Figure 9D), the very poor bioavailability of curcumin would not seem to justify the in vivo pharmacological responses against CF, which are indeed observed (Supplementary Table S1). The curcumin concentrations used to investigate the effects on CFTR function for in vitro studies range from a few micromolar to 60 μM (the highest soluble concentration [14]), which are higher than the submicromolar physiological concentrations normally achieved by this compound. Nevertheless, curcumin administration to patients with cystic fibrosis produced remarkable benefits in two patients carrying respectively homozygous F508del and F508del/G1061R CFTR mutations (3-year treatment) [17] and in a randomized control-controlled clinical involving 20 children diagnosed with CF (6-month treatment; type of mutations is not reported) [25]. In an open-label intervention study, Berkers et al. [40] examined the effects of 8-week treatment with curcumin plus genistein, ivacaftor, and ivacaftor plus genistein on patients carrying at least one S1251N mutation. In the case of the curcumin plus genistein combination, the authors observed no clear clinical improvements except a small but statistically significant change in sweat chloride concentration and airway resistance. Plasma samples from the patients treated with the curcumin/genistein combination failed to induce swelling responses in intestinal organoid assays. In contrast, organoid assays employing a combination of the pure drugs (10 μM genistein plus 50 μM curcumin) produced results in line with previously published results [15]. Berkers et al. [40] deemed the low plasma concentrations as responsible for the modest effects of curcumin observed in vivo against CF contrasting with the important effects found in vitro. However, the authors of the above open-label intervention study did not report the possible influence of piperine, which was included in the curcumin supplements given to patients. Piperine blocks intestinal Cl^−^ secretion (IC_50_ of ∼5–10 μM) by inhibiting CFTR, Ca^2+^-activated Cl^−^ channels and cAMP-activated basolateral K^+^ channels and is used traditionally for its antisecretory activity [41]. It must be clarified whether the administration of piperine to patients with CF is counterproductive and/or negatively affects the outcome of clinical studies on CFTR modulators.

Since curcumin has a pleiotropic nature and acts by distinct mechanisms, either indirectly or directly on CFTR, possibly employing distinct binding sites on CFTR, it appears that this compound, even at very low physiological concentrations, is capable of activating at least some of the multiple mechanisms that can improve CFTR function. In addition to the benefits of curcumin on multiple pathways not directly involving the binding to CFTR, the evidence of CFTR modulation in vitro and the correlated improvements of CF symptoms in vivo is substantial (Supplementary Table S1). The apparent discrepancy between the scarce physiological concentrations achievable by curcumin and its observable effects against CF, even in vivo, might be explained by the curcuminoid consensus binding site proposed in this study. The closed-to-open conformational conversion of CFTR channels causes the transformation of the consensus binding site from a solvent-exposed groove to a closed protein cavity. This produces a trapping effect on pre-bound curcumin molecules, which is not taken into account by binding free energy calculations estimating only the instantaneous interaction between the ligand and neighboring protein residues. Upon channel opening, the ligand becomes confined in a cavity, floating inside it without an escape route to the bulk solvent, thus perpetuating the interactions and sustaining the open conformation indefinitely. This explanation is consistent with the observation that curcumin irreversibly activates CFTR with a mechanism not involving chemical cross-linking [42]. Finally, only tiny amounts of curcumin bind CFTR in vivo, but once the channels open, the compound may persistently sustain its activation. Thus, curcumin can work at very low physiological levels. A possible analogy is represented by the binding of ATP cofactors, which are squeezed into a cavity in the protein upon the dimerization of NBD1 and NBD2. Despite their calculated free binding energy being significantly lower than those of the curcuminoids or drugs (Figure 9D), the detachment of these cofactors from CFTR requires the intrinsic ATPase activity of the channel. Of note, none of the binding sites of ivacaftor, lumacaftor, elexacaftor, and other CFTR drugs so far disclosed by cryo-EM exploits the curcuminoid binding site. Given the pleiotropic nature of curcumin, it cannot be excluded that curcumin might also bind other CFTR sites and activate the channels with bound ATP cofactors. Indeed, different groups have proposed that drugs modulating CFTR might work through multiple binding mechanisms [30,38].

To address which is the most active curcumin tautomer in this model of ligand binding to CFTR, the comparison of their interaction energies with CFTR (Figure 9A,B), as well as their binding affinities (Figure 9C), shows that keto-enol curcumin binds more strongly than diketo curcumin to both closed and open channels. While this might not suffice to endow keto-enol curcumin with CFTR potentiation capability exclusively, it must be noticed that only this curcumin tautomer has an electrophilic α,β-unsaturated carbonyl group (Figure 3), a moiety susceptible to Michael addition, and indeed, covalent adducts, typical of this reaction, between curcumin and CFTR have already been reported [42]. The Michael addition involves the attack at the β carbon by nucleophilic groups such as the side chain of cysteine and serine (deprotonated forms) or lysine and histidine (unprotonated forms). It can be seen that while in the closed CFTR, only one potential nucleophile, Ser492, resides sufficiently close to the α,β-unsaturated carbonyl group of curcumin (Figure 7), in the open CFTR additional nucleophilic residues become available owing to the NBD1-NBD2 association (Figure 8). Among these, Cys1344, His1348, and Lys1351 place their nucleophile side chains in direct contact with the electrophilic group of curcumin. In particular, the cysteine residue, in its anionic form, is known as the most potent Michael nucleophile among amino acids. Of note, Cys1344 deprotonation to a thiolate can be assisted by the adjacent Asp1341. The possibility that curcumin in the consensus binding site may undergo Michael addition reactions support the curcumin-induced CFTR cross-linking observed in a previous study and further suggests that keto-enol curcumin is the curcumin tautomer promoting CFTR activation. Together with the poor bioavailability, the Michael addition reaction might be another factor limiting curcumin CFTR potentiation efficiency. To delve into the role of Michael addition in curcumin-induced cross-linking of CFTR, a curcumin analog, BSc3596, which bears a cyclic 1,2-oxazole group instead of the reactive β diketone moiety, was employed, showing potent activation of the wild-type CFTR, G551D-CFTR, and Delta1198-CFTR without causing CFTR cross-linking [42]. The cyclic oxazole moiety also constrains BSc3596 into a more rigid linear shape, placing the two 2-methoxyphenol groups far apart, which could explain the potency of this curcuminoid. Somehow, a linear molecular geometry also characterizes keto-enol curcumin, which further suggests that this curcumin tautomer is a more efficient CFTR activator than diketo curcumin.

Provided that CFTR mutations do not affect the binding site of curcuminoids, it is possible that these compounds can stimulate a large array of malfunctioning channel mutants, especially those with impaired dimerization of the NBDs. The model for curcuminoid binding proposed in this study can be used in the design of CFTR modulators for these particular mutants.

## 4. Materials and Methods

### 4.1. Docking

The geometry of the ligands employed in docking (diketo curcumin, keto-enol curcumin, and BSc3596 was optimized with the semiempirical method RM1 (below a 0.01 kcal mol^−1^ Å^−1^ gradient) with HyperChem v8.0 (Hypercube, Inc., Gainesville, FL, USA). The ligand connectivity, atom, and bond types were generated with SPORES v1.3 [43]. The protein docking targets consisted of multiple conformer ensembles of the closed CFTR, the open CFTR, and the open CFTR lacking NBD2 (open CFTR-ΔNBD2), each represented by twelve conformers. Each ensemble included a PDB structure relevant to the above protein states (PDB 6MSM for the open CFTR and open CFTR-ΔNBD2; PDB 5UAK for the closed CFTR), its energy minimized structure and ten MD-derived conformers (please see the MD simulations section below). The R domain peptide fragment and the ATP molecules present in the electron microscopy PDB structures were not taken into account in the docking. Overall, the binding pose of the 3 curcuminoid structures was searched on 3 × 12 protein conformers for a total of 108 distinct docking searches (schematized in Figure 3). Prior to docking, the 36 protein targets were structurally aligned relative to the conformationally invariant regions highlighted in Figure 2 for straight binding pose comparison. The docking search space was set within 33.0 Å from the NBD1 center, which enabled full binding exploration within this domain, plus the ICL1 and ICL4 portions laying in contact with it (Figure 3). Docking was carried out with PLANTS (v1.2) [44]. Each docking search was set to collect 5000 non-redundant ligand poses (RMSD > 2.0 Å) ranked with the chemplp scoring function, and the top ten scoring poses resulting from each docking search were subjected to further analysis.

### 4.2. MD Simulations

MD simulations on CFTR protein structures were performed to generate the docking targets consisting of three CFTR conformer ensembles representing the channel in the different functional states, open, open CFTR, open CFTR-ΔNBD2, and closed CFTR) and also to assess the stability of curcuminoids bound to the closed and open CFTR. Specifically, the electron microscopy PDB structure 6MSM was used to produce both the open wild type CFTR and open CFTR-ΔNBD2 ensembles (the latter obtained by deleting the protein residues from Asp1202 to Pro1451 and assuming it presented the same fold as the parent structure) and the electron microscopy PDB structure 5UAK for the closed CFTR ensemble. The MD conformers of CFTR used in the docking ensembles were taken at 1 ns time intervals starting from time 1 ns. The curcuminoid-CFTR complexes employed in the MD simulations were prepared by placing the ligands on the closed CFTR (PDB 5UAK) and open CFTR (PDB 6MSM) structures according to the consensus binding pose obtained with the docking procedure (please see Section 2). The ligands in the binding site were geometry-optimized before the MD simulations. The bound ATP ligands and the partially solved R domain were removed from structures prior to all simulations. The molecular systems were solvated in explicit water (TIP3P water model), adding Na^+^ and Cl^−^ ions to achieve electroneutrality and ionic strength of 0.1 mol/L, and energy minimized for 20,000 steps. The MD simulations were performed with NAMD (v.2.14 with CUDA GPU Acceleration) [45,46] under periodic boundary conditions using the CHARMM22 protein force field [47], including the CMAP correction and the CHARMM general force field (CGenFF) [48] for ligands as implemented in SwissParam [49]. The temperature was maintained at 310 K with a Langevin thermostat using a damping coefficient of 1 ps^−1^. The integration step was 1 femtosecond, and flexible bonds were adopted. Short-range interactions were computed every 1 time step, long-range electrostatic interactions every 2 time steps, applying 10 Angstrom switching distance, 12 Angstrom cut-off, and 13.5 Angstrom pair list distance. The atomic coordinates were recorded every 5000 femtoseconds. The interaction energies were calculated with the NAMDEnergy plugin (v1.4) of VMD (v1.9.3) [50]. Binding free energy was calculated with KDEEP [51]. In the case of the curcuminoids described in this study, the binding affinity calculations were made on their complexes with CFTR inclusive of the hydrogen atoms as derived from the MD simulations. In the case of the complexes of drugs with CFTR available from PDB cryo-EM structures, the binding affinity calculations were made after adding and optimizing hydrogens. Molecular graphics were made with PyMOL (v0.99) (https://pymol.org).

## 5. Conclusions

In this study, the authors propose that curcumin binds the closed CFTR near the interface formed by NBD1, ICL1, and ICL4. When the channel opens spontaneously with pre-bound curcumin, this ligand, in addition to maintaining its contact with NBD1/ICL1/ICL4, simultaneously engages in interaction with NBD1, ICL2, and ICL3, finally bridging all protein domains and stabilizing the active channel configuration. This mechanism might explain curcumin’s ability to potentiate CFTR mutants characterized by defects in ATP binding and/or intramolecular dimerization of the NBDs.

Experimental validation is needed to confirm whether this binding mode reproduces the native binding of curcuminoids. Nonetheless, this model highlights a potentially exploitable mechanism of activation that can be triggered by drugging the NBD1/ICL1/ICL4 interface and provides a basis for the design of novel activators of CFTR mutants characterized by subnormal NBD dimerization capability.

## Data Availability

The data presented in this study are available on request.

## Supplementary Materials

The following supporting information can be downloaded at: https://www.mdpi.com/article/10.3390/ijms25010552/s1.
