# Supplementary material for: Exploring the Mechanism of Activation of CFTR by Curcuminoids: An Ensemble Docking Study
Source: Int J Mol Sci. 2023 Dec 31;25(1):552. doi: 10.3390/ijms25010552 (PMC10778693; doi:10.3390/ijms25010552)
Supplement: Supplementary file 1 [file ijms-25-00552-s001.zip › ijms-2733827-supplementary.pdf]

# Exploring the Mechanism of Activation of CFTR by Curcuminoids: An Ensemble Docking Study

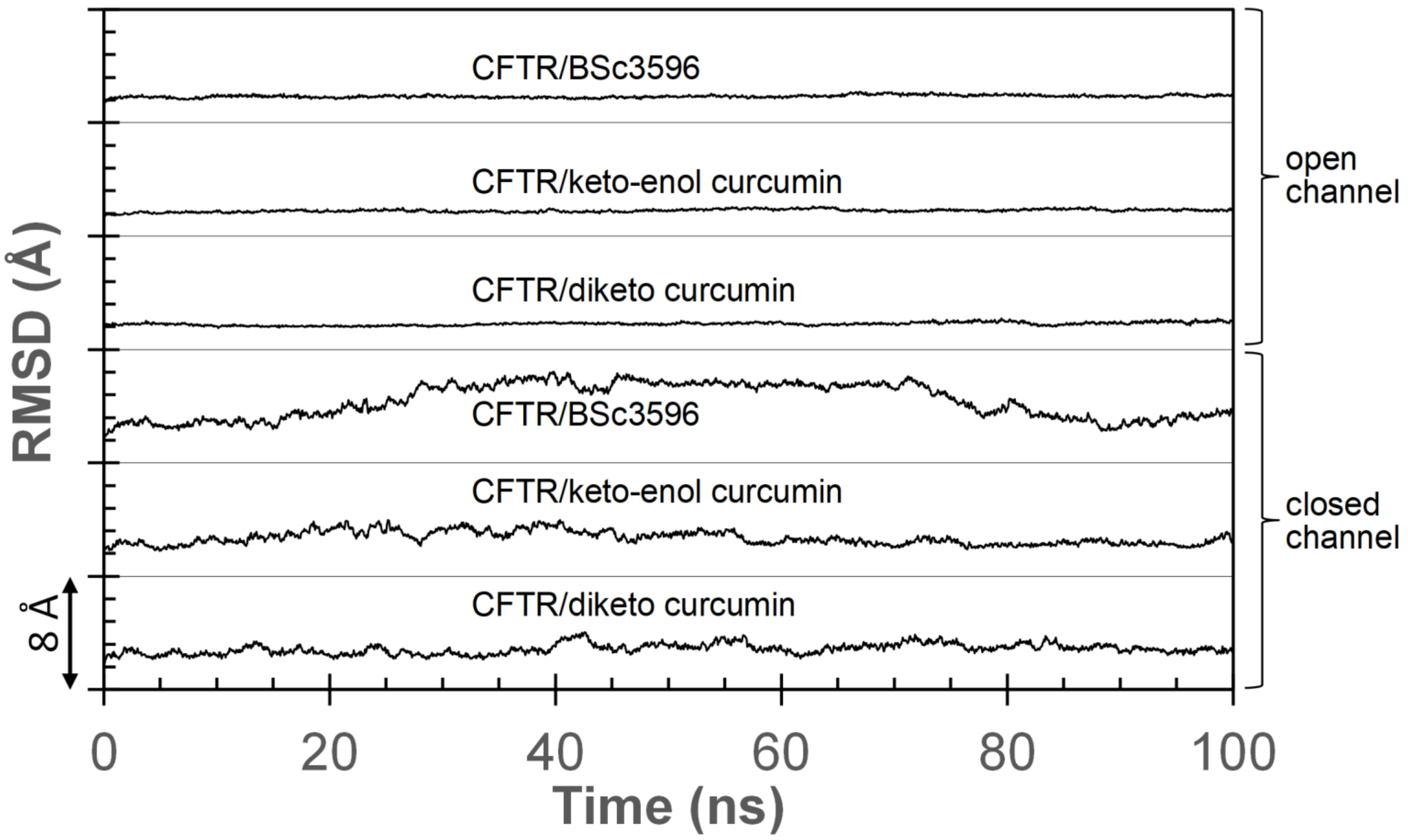


Figure S1: RMSD traces of the MD simulations.

**Supplementary Table S1.** Effects of curcumin related to cystic fibrosis observed in *in vitro*, *in vivo* and in clinical studies.

| *Curcuminoid preparation* | *Curcuminoid dosage or concentration* | *Type of study* | *Investigated CFTR mutation(s)* | *Relevant observations* | *References* |
|---|---|---|---|---|---|
| Curcumin (Sigma, catalogue number C7727) | 15 or 45 mg/kg/day, orally for 3 days | *In vivo* (mice) | Homozygous F508del | Correction of abnormal nasal potential difference to almost baseline value. | 16 |
| Curcumin root powder from an Asian market.<br><br>95% root curcumin | Case study 1:<br>3.6-8 g/day (~69 to 154 mg/kg/day) orally<br><br>Case study 2:<br>8 g/day (~114 mg/kg/day) orally. In both case 1 and 2, curcumin treatment was going on for three years at the time of the report. | Case reports | Case study 1:<br>45-year-old, ≈ 52kg weight (BMI 21), female with homozygous F508del.<br><br>Case study 2:<br>41-year-old, 70kg weight (BMI 22), male with F508del/G1061R | - The patient in case 1 reported improved sputum, resolution of hemoptysis, arterial hemoglobin oxygen saturation rising above 88% with exercise, and no need of further antibiotics.<br>- The patient in case 2 reported noticeably improved exercise tolerance and sputum production. Both patients did not note adverse effects. | 17 |
| Curcumin used for most experiments was from Sigma (catalogue number C7727; >94% curcuminoid content with >80% curcumin).<br><br>BSc3596 preparation is described in ChemMedChem. 2008;3:165-72. | Curcumin 7-50 μM<br><br>BSc3596 0.5-30 μM | *In vitro* | Δ1198-CFTR, G551D | Curcumin and BSc3596 irreversibly activate wild-type CFTR, G551D-CFTR, and Δ1198-CFTR. Curcumin can also cause CFTR cross-linking. | 42 |
| Curcumin (Sigma-Aldrich) stored as a 30 mM stock in DMSO at -20 °C and dissolved in the bath solution just before use | 5 μM and above | *In vitro* | G551D | In the lower concentration range, curcumin (5 μM) and genistein (10 μM) synergistically restore the gating defect of G551D-CFTR.<br><br>At higher concentrations, CFTR potentiation induced by one compound becomes additive to the potentiation induced by the other compound, suggesting distinct mechanisms for the two compounds. | 14 |
| Unspecified | 50 μM curcumin | *In vitro* | S813A (abrogates S813 phosphorylation by PKA), Y161A, K166H, F1078A, and R1066H | The single mutations suppress curcumin-induced CFTR potentiation. | 28 |

| | | | | | |
|---|---|---|---|---|---|
| Curcumin (Fluka; 08,511-10MG, stock solutions prepared in DMSO and stored at -80 °C for a maximum of 6 months). | >12.5 μM | *In vitro* (organoids from patients with CF) | S1251N, G551D, and homozygous F508del | Synergistic reparation of CFTR-dependent forskolin-induced swelling by combinations of ivacaftor, genistein and curcumin. | 15 |
| Curcumin nanoparticles (Sina curcumin 80; Minoo Pharmaceutical Co). | 3 x 80 mg/day (~9 mg/kg/day), orally for 6 months | randomized control-controlled clinical study on children aged between 5 and 18 years old | Not specified | Significant positive effects on body weight and emotional functioning scores, and a trend towards improvement in physical and school functioning scores. | 25 |
| Combination of curcumin and genistein.<br><br>The curcuma preparation was AOV 811, which contains curcuma longa root extract (minimum 95% curcuminoids; 600 mg/capsule)<br><br>and<br><br>piper nigrum extract (minimum 95% piperine; 5 mg/capsule).<br><br>Genistein preperation was AOV 805.<br><br>The preparations were produced by the company AOV (part of Atrium Innovations INC). | Curcumin dose was between 102.9 and 138.5 mg/Kg/day (median dose = 7200 mg/day corresponding to ≈ 102.9 to 133.3 mg/kg/day based on patients' weights).<br><br>Genistein dose was between 3.3 and 5.0 mg/Kg/day.<br><br>Piperine dose was between 29 and 96 mg/day.<br><br>The supplements were given in 3-4 doses, orally for 8 weeks.<br><br>The measured median curcumin plasma concentration was:<br><1 μg/L (curcumin without its metabolites)<br>387 μg/L (curcumin plus its metabolites). | Open label intervention study (it was also measured the response of intestinal organoids to either the pure compounds or the plasma of treated patients) | Patients with at least one S1251N mutation | Study 1 (NTR4585) was an eight-weeks open label intervention study with a combination of curcumin (AOV 811 curcuma longa) and genistein (AOV 805 genistein).<br><br>Study 1 showed no clear functional effects related to CF except a small but statistically significant change of sweat chloride concentration and airway resistance. However, the curcumin preparation employed in the study also contained piperine. The effects of administering piperine to patients with CF have not been investigated. Since piperine is a CFTR inhibitor (see reference 27), this compound may adversely affect CF. | 26 |
